# Supplementary material for: Estimation of the Uncertainties Related to the Measurement of the Size and Quantities of Individual Silver Nanoparticles in Confectionery
Source: Materials (Basel). 2019 Aug 22;12(17):2677. doi: 10.3390/ma12172677 (PMC6747558; doi:10.3390/ma12172677)
Supplement: Supplementary file 1 [file materials-12-02677-s001.pdf]

materials

Supplementary Materials

Estimation of the Uncertainties Related to the Measurement of the Size and Quantities of Individual Silver Nanoparticles in Confectionery

S1. Single-Particle ICP-MS/MS Instrumentation

An Agilent 8800 ICP-MS/MS (Agilent Technologies) was used for data acquisition in time-resolved analysis mode. The ^{107}Ag signal intensity was recorded at 3 ms dwell times during 60 s. The peristaltic pump was set to 0.15 rps, which translates to a sample flow rate of approximately 0.47 mL/min. The sample flow rate was accurately determined on a daily basis by weighing the amount of water delivered over an 8-minute period. To increase the sensitivity of the spICP-MS analyses, instrument tuning was optimized for ^{107}Ag by adjusting, amongst others, sample depth and carrier gas flow rate. Instrument calibration was performed daily by analyzing certified dissolved Ag standards (TraceCERT® silver standard for ICP, 994 ± 3 mg/L) at 1 and 2.5 µg/L and a blank (UPW) in time-resolved analysis mode. The Ag intensity for each standard solution was averaged over the entire length of analysis (60 s). The transport efficiency was determined daily according to the particle frequency method [1] by means of the reference material NIST-8012 (gold nanoparticles, nominal size 30 nm) or 30-nm gold nanoparticles from nanoComposix, both at a concentration of 12.5 ng/L under the same instrumental conditions as the samples. The transport efficiency was calculated as the ratio of the number of particles detected by spICP-MS and the theoretical particle number. It varied between 4.4% ± 0.4% and 7.4% ± 0.4% among the different validation exercises. The instrument parameters and operational conditions are given in Table S1. Each analysis was followed by a 40 s rinse with HCl 5% (*v*/*v*) and a 160 s rinse with HNO_3 4% (*v*/*v*) or a 160-s rinse with a mixture of 1% (*v*/*v*) HCl (34–37%), 1% (*v*/*v*) HNO_3 (67–69%), and 0.1% (*w*/*v*) Triton X-100. After the rinsing procedure, ultrapure water was analyzed to verify that no carry-over of Ag from the previous sample occurred. Data were csv files to be processed in Microsoft Excel.

Table S1. Settings used for spICP-MS measurements with ICP-MS/MS.

Instrument Parameter	Operation Settings
Nebulizer	Micromist
Spray chamber	Quartz, double pass
Sampler and skimmer cones	Nickel
RF power (W)	1550
Plasma gas flow (L/min)	15
Auxiliary gas flow (L/min)	0.90
Carrier gas flow (L/min)	1.09
Sample flow rate (mL/min)	0.47 ± 0.02
Dwell time (ms)	3
Sampling time (min)	1
Transport efficiency (%)	4.4 ± 0.4–7.4 ± 0.4
Monitored element	Ag
Isotope (amu) monitored at Q1–Q2	107–107
Elemental composition of the target particle	Ag
Density (g/cm^3)	10.49
Mass fraction particle/analyte	1
Ionization efficiency (%)	100

S2. Determination of Additional Performance Characteristics for Single-Particle ICP-MS

Selectivity—Selectivity is defined as "the extent to which other substances interfere with the determination of a substance according to a given procedure" [2]. "Other substances" may refer to other nanoparticles as well as to other materials present in the sample and, in the case of ICP-MS analyses, interferences.

Selectivity against matrix components—the main ingredient of the food products containing E174 are carbohydrates, which represent about 75% (*w*/*w*) of the ingredients in the silver-coated chocolates and 98% (*w*/*w*) in the silver pearls. Therefore, the selectivity of the spICP-MS method to characterize AgNP against the presence of carbohydrates was tested by means of a silver nanoparticle standard solution (nanoXact 20 nm, nanoComposix, San Diego, CA, USA). Respectively 0, 1, and 2 g of sugar were added to 0.25 mL of the nanoXact solution and made up to 25 mL with 0.125 mL ethanol (96%) and 24.625 mL BSA solution (0.5 mg/mL). The addition of sugar did not have an effect on the median ESD, particle number, or particle mass concentration, taking into account the uncertainty on the repeatability of the analysis (Figure S1).

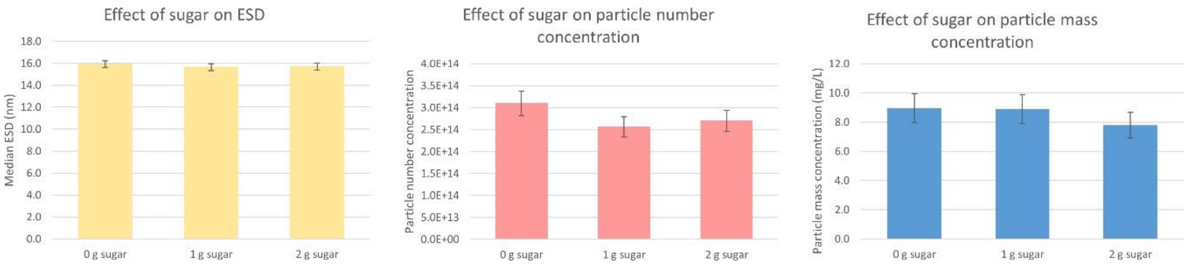

Figure S1. Effect of sugar on the median equivalent spherical diameter (ESD), particle number, and mass concentration. Error bars represent the repeatability uncertainty (k = 2; see further).

Selectivity against other types of nanoparticles—spICP-MS is, by virtue of the highly selective mass spectrometer, a selective method towards nanoparticles of different composition. The ICP-MS quantification method at *m*/*z* 107 is highly specific for AgNP, hence the selectivity against other types of nanoparticles is not an issue for these spICP-MS analyses.

Applicability of different varieties of the same type of nanoparticles—to cover the applicability of the sample preparation and spICP-MS methodology to different types of E174 and food products containing E174, repeatability and reproducibility experiments were performed on two different types of E174 (2-mm silver flakes (Ag-005) and 8-cm silver leaves (Ag-008)) and two different types of food products containing E174 (silver-coated chocolates, also known as mini "sugar beans" (Ag-P-003) and silver pearls containing mainly sugar and wheat flour/corn meal (Ag-P-002)).

Robustness/ruggedness—The "ruggedness" ("robustness") of an analytical procedure is a measure of its capacity to remain unaffected by small but deliberate variations in method parameters [3] (Eurachem, 2015). Table S2 gives an overview of the relevance of selected parameters that may affect the spICP-MS procedure and the measures that have to be taken to control these parameters.

Table S2. Relevance of selected parameters with regard to ruggedness and measures to control these parameters.

	Relevance	Control Measures
	Laboratory Equipment	
Balances	Relevant: dilutions are prepared gravimetrically.	Daily verification and yearly maintenance.
Glassware and plasticware	Relevant: contamination has to be avoided.	Only clean glassware should be used. For AgNP analyses, all glassware and plasticware has to be cleaned by soaking it in nitric acid

		(10%), rinsing twice with double-distilled water and once with UPW.
ICP-MS	Relevant: sensitivity determines the limit of detection.	Daily tuning of the ICP-MS, yearly maintenance of the apparatus.
ICP-MS tubing	Relevant: contamination and carry-over of previous samples has to be avoided.	Rinsing with 4% (*v*/*v*) HNO_3 (67–69%) for 40 s, followed by a 155 s rinse with 1% (*v*/*v*) HCl + 1% (*v*/*v*) HNO_3 (67–69%) + 0.1% (*w*/*v*) Triton X-100, analysis of UPW samples after the rinsing procedure to verify that no carry-over occurs.
	Samples and Products	
UPW	Not relevant if produced daily.	
Standards	Relevant: nanoparticles in dispersions may become unstable over time; ionic standards are not prepared in an acid environment.	Daily fresh preparation of calibration standards. Nanoparticle stock solutions may be used over a longer period. A drop in the transport efficiency indicates when the nanoparticle dispersion is no longer fit for use.
Samples	Relevant: nanoparticles in the prepared samples my become unstable.	Samples have to be measured immediately after sample preparation and dilution.
	Method Principles	
Assumptions about shape and composition	Relevant: spICP-MS assumes a spherical geometry for the nanoparticles when recalculating the measured mass into a volume.	The actual shape of the nanoparticles is ideally verified by TEM. Other formulas to calculate the volume of the particles may be used.
	Environmental Conditions	
Room temperature	Not relevant: daily tuning of the ICP-MS signal optimizes the signal as a function of the daily conditions; calibration occurs within the same time frame as the unknown samples, hence under the same environmental conditions.	

Calibration and linearity—For the analysis of AgNP by spICP-MS, two types of calibration are necessary: a particle calibration to determine the transport efficiency (see S1), and a mass calibration to relate the ICP-MS signal intensity to the mass of the particle.

The mass calibration was performed with UPW (blank) and ionic silver standard solutions diluted in UPW. These mass calibration standards were analyzed by spICP-MS (i.e., in time-resolved analysis mode), and a regression coefficient was calculated between the average response in the calibration standard (in cps) and the nominal Ag concentration (µg/L). As the signal for Ag in UPW is not a continuous signal, it was chosen not to use the UPW standard but instead only use the silver standards and force the regression function through zero. Examples of regressions obtained over the validation period are given in Table S3 and Figure S2. The measurements yielded linear curves for intensity versus silver concentration. The slopes on the different days laid in a similar range but varied between days, necessitating a daily calibration.

Table S3. Examples of regression slopes and R^2 values for regression curves with and without forcing through zero.

Date	Full Regression		Forced Through Zero Regression	
	R^2	Slope (cps/(µg/L))	R^2	Slope (cps/(µg/L))
08/03/2018	0.9965	279,060	0.9981	286,212
16/03/2018	1.0000	275,999	1.0000	276,634
05/06/2018	0.9998	346,707	0.9999	349,069
07/02/2019	0.9999	393,287	0.9999	395,545
14/02/2019	0.9999	330,741	0.9999	329,527
07/03/2019	1.0000	262,564	1.0000	262,063

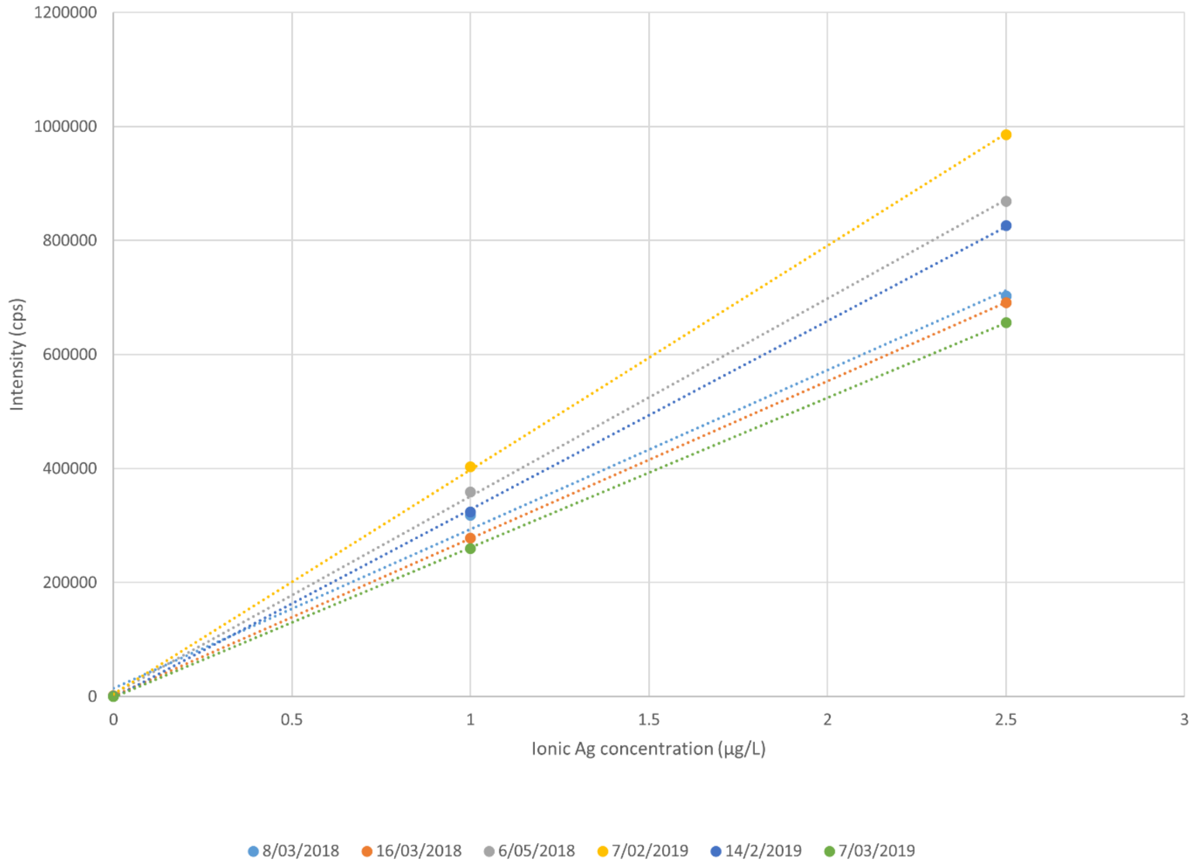

Figure S2. Examples of calibration curves relating signal intensity to Ag concentration. The dotted lines represent the linear regression lines through the three calibration levels (full regression).

S2.1. Limit of Detection and Quantification (LOD and LOQ) and Working Range

Particle size—It is common in ICP-MS analysis that even when blank samples (e.g., UPW) are analyzed, a low-intensity signal is detected due to dark noise (the detector registers a signal even without an ion beam) and shot noise (undesirable photons, electrons, or ions hitting the detector [4], 2013). If one count due to noise is detected in a dwell time of 3 ms, this will result in a signal intensity of 333.33 cps. Hence, when analyzing UPW, signal peaks at 333.33 cps, 666.66 cps, 1000 cps, etc. are typical. The smallest particle that can be detected in single-particle mode depends on the distribution of this noise signal. Concentration detection limits in conventional ICP-MS are usually stated in terms of standard deviation of the background signal. The size detection limit for spICP-MS may similarly be expressed in terms of standard deviation of the noise signal, σ_{noise}. To discriminate between the noise signal and particle events, it was chosen to work with a probability of erroneously detecting a particle when it is truly not present (Type I error) of 1 per 33,000 counts, which in practice means less than one during the 60 s measurement time. Hence,

$$LOD_{intensity} = \mu_{noise} + 4\sigma_{noise} \quad (1)$$

(remark: a Gaussian distribution was assumed, although a Poisson distribution was probably followed).

To determine σ_{noise}, an iterative algorithm was applied based on a detection limit 4σ. The data points exceeding $\mu + 4\sigma$ were considered particle signals and were removed. These calculations were repeated multiple times until no further data points were removed. The $LOD_{intensity}$ was then recalculated into particle size by applying typical, minimal, and maximal values for the sample flow rate (V), transport efficiency (η), and ICP-MS response (Table S4). As these parameters varied on a daily basis, the particle size detection limit was also a variable parameter.

Table S4. Values used to recalculate signal intensity to size.

	Typical Value	Minimal Value	Maximal Value
Element density (g/mL)	10.49	–	–
Dwell time (s)	0.003		–
ICP-MS response of standard ion (cps/(µg/L))	298,500	395,500	248,300
Flow rate (mL/min)	0.47	0.43	0.50
Transport efficiency (−)	0.053	0.040	0.079

The $LOD_{intensity}$ was 1500 cps. Applying typical values for sample flow rate, transport efficiency, and ICP-MS response, this resulted in a "typical" size LOD of 9 nm. The size LOD decreases if the flow rate or transport efficiency decrease, or if the ICP-MS response increases. By applying minimal and maximal values for these three parameters, the size LOD of the spICP-MS analysis of AgNP can theoretically range between 7.3 and 11 nm.

In practice, AgNP are rarely present in a sample without the co-presence of ionic Ag, which may interfere with the detection of AgNP, as both signals might overlap. The "limit for particle detection" (i.e., the signal intensity that separates the background signal caused by Ag ions or incomplete particle events from the particle signal) has to be determined sample-by-sample, as it depends on the specific conditions of the sample (ion concentration, AgNP size, AgNP number concentration, dilution factor, etc.). The limit for particle detection can be derived by the iterative algorithm described above, visually on a pulse intensity frequency histogram, or by expert judgement. The limit for particle detection equals the quantification limit, and for NM-300K was in the range of 11–13 nm.

Particle number concentration—The limit of detection for the particle number concentration (C_p) in dispersions diluted for spICP-MS analysis was determined by the minimal number of particles to be detected per minute ($q_{p,\,min}$) in order to get a reliable distribution. The minimal number of detected particles was set at 200/min. The limit of detection for particle number concentration was calculated as:

$$LOD\ C_p = q_{p,\,min}/(\eta.V/1000), \quad (2)$$

in which $q_{p,\,min}$ is the minimal number of particles to be detected per minute (200/min), η is the transport efficiency, and V is the flow rate (mL/min). The values for flow rate and transport efficiency in Table S4 were used to calculate the particle number concentration LOD, which ranged between 0.5 and 1.2×10^7 particles/L in the diluted dispersion ready for spICP-MS analysis.

Particle mass concentration—The limit of detection for the mass concentration (C_m; ng/L) in dispersions diluted for spICP-MS analysis, is determined by the particle number LOD and the size of the particle. The limit of detection for mass concentration is calculated as:

$$LOD\ C_m = LOD\ C_p\ .\ m_p = LOD\ C_p\ .\ \varrho.\ 10{-}12.\ \pi\ .\ d^3/6, \quad (3)$$

with m_p the particle mass, ϱ the particle density, and d the particle diameter. Figure S3 represents the range of mass concentration LODs for silver particles as a function of their size.

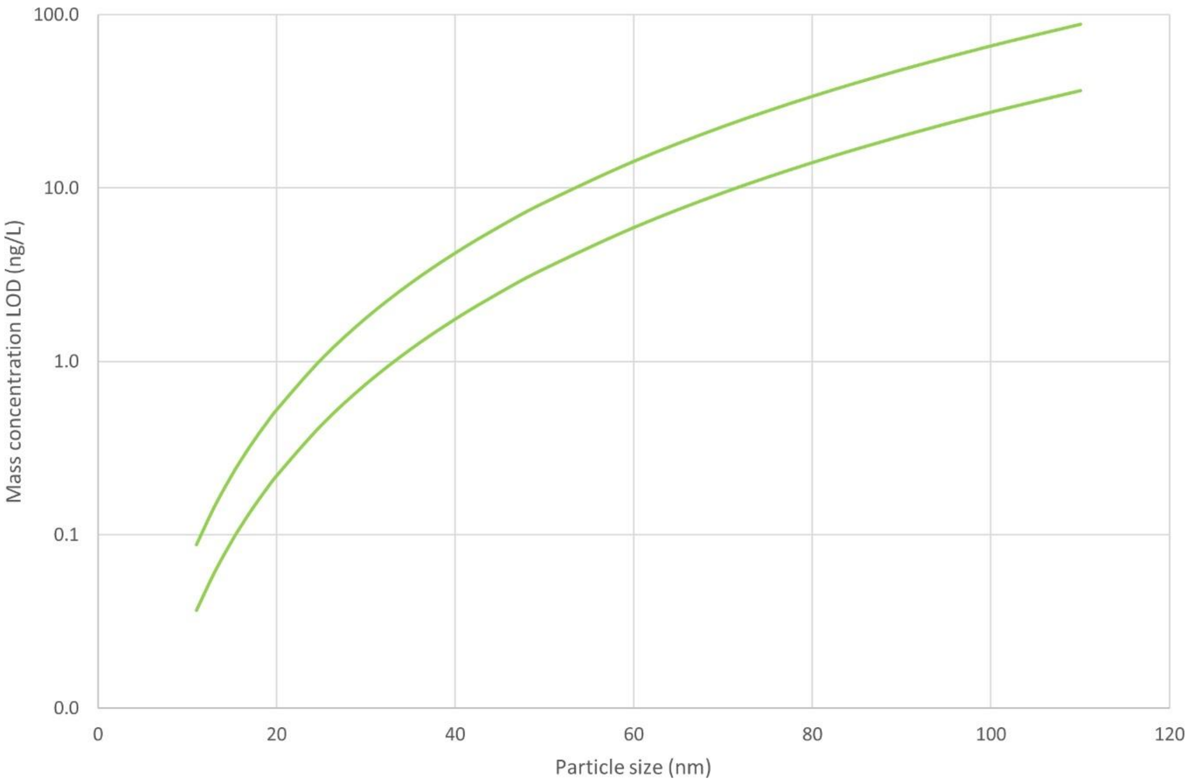

Figure S3. Mass concentration detection limit for AgNP in dispersions diluted for spICP-MS analysis as a function of the size of the particle. The two lines represent the detection limit boundaries, calculated with minimal and maximal values for the particle number concentration LOD.

Working range in polydisperse dispersions—Ideally, the number of peaks detected in a diluted dispersion should range between 200 and 2000, although 3000 peaks gave no different results. The number of peaks depends on the size of the particles and their mass concentration:

$$C_m = C_p \,.\, m_p = C_p \,.\, \varrho.\ 10–12.\ \pi \,.\, d^3/6. \quad (4)$$

Hence in polydisperse dispersions (i.e. dispersions in which particles of different size distributions are present) with equal mass concentrations for the nanoparticles of different sizes, the working range of the particle size is limited by the mass concentration, the flow rate of sample introduction, and the transport efficiency (Figure S4). In polydisperse dispersions of equal particle number concentration, there is no maximum mass or particle number concentration that can be measured, as the dispersions can always be further diluted to obey the single-particle principle.

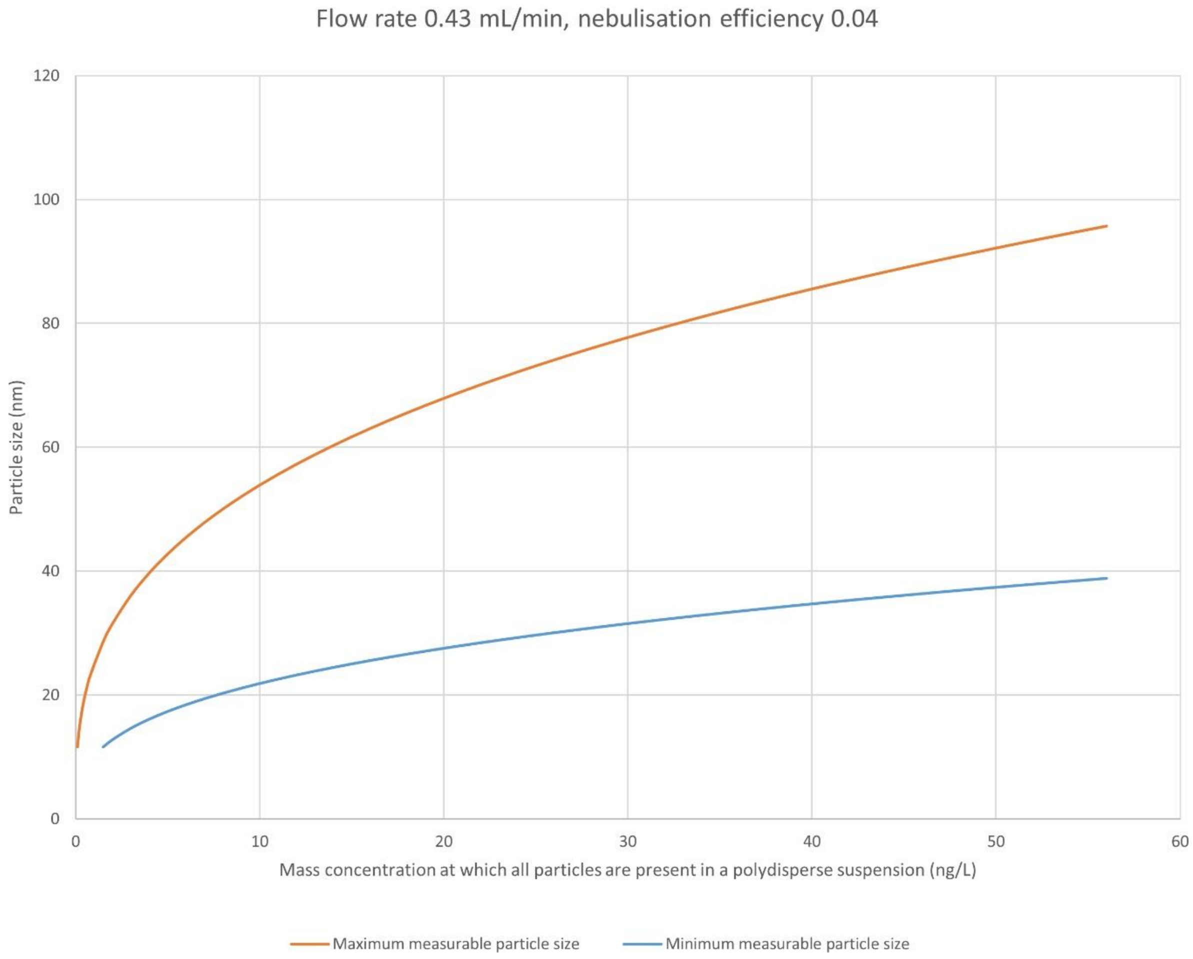

Figure S4. Example of the working range of particle size in polydisperse dispersions of AgNP at equal particle mass concentration as a function of the mass concentration, flow rate, and transport efficiency. The working range is presented by the area between both curves.

S3. Validation of the Measurement of the Minimal External Dimension of the Constituent Particles of NM-300K by TEM Imaging Combined with Image Analysis by the ParticleSizer Software

S3.1. Definitions and Abbreviations

S3.1.1. Definitions

Pixel size: the size of one pixel in an EM image (in nm).

Particle: minute piece of matter with defined physical boundaries [5].

Aggregate: particle comprising strongly bonded or fused particles where the resulting external surface area may be significantly smaller than the sum of calculated surface areas of the individual components [6,7].

Agglomerate: collection of weakly bound particles or aggregates or mixtures of the two where the resulting external surface area is similar to the sum of the surface areas of the individual components [6,7].

Feret-min: the minimum distance between the two parallel tangents touching the particle outline in all directions.

ECD: Equivalent circle diameter

$$2\sqrt{\frac{Area}{\pi}}. \quad (5)$$

Aspect Ratio:

$$\sqrt{\frac{\text{Long Side Minimum Bounding Rectangle}}{Short\ Side\ Minimum\ Bounding\ Rectangle}} \quad (6)$$

Solidity

$$\sqrt{\frac{\text{Area}}{Area\ Convex\ Hull}}. \quad (7)$$

The above-described definitions of the physical particle parameters are shown following [8] and [9].

General concepts and terms used in the European Commission's definition of a nanomaterial are described in [10].

S3.1.2. Abbreviations:

ECD: equivalent circular diameter
EM: electron microscopy
ERM: European reference material
Feret-min: minimum Feret diameter
NM: nanomaterial
nm: nanometer
NP: nanoparticle
sd: standard deviation
SOP: standard operating procedure
TEM: transmission electron microscopy
u_{cal}: uncertainty associated with calibration
$u_c(x)$: combined uncertainty
U_{cx}: (expanded) measurement uncertainty (k = 2)
u_{day}: uncertainty due to day-to-day variation
u_{IP}: uncertainty associated with within-lab intermediate precision
u_r: uncertainty associated with repeatability
u_{tr}: uncertainty associated with trueness
$u_{t\,CRM}$: uncertainty associated with trueness of the CRM
Xm: mean

S3.2. Scope of the Validation Study

The aim of this validation study was to validate the SOP entitled "Measurement of the minimal external dimension of the constituent particles of NM-300K by TEM imaging combined with image analysis by the ParticleSizer software". This SOP is based on the combination of three general SOPs for TEM specimen preparation "Preparation of EM-grids containing a representative sample of a dispersed NM", TEM imaging "Transmission electron microscopic imaging of nanomaterials", and image analysis "Measurement of the minimal external dimension of the constituent particles of particulate materials from TEM images by the NanoDefine ParticleSizer software". The ParticleSizer automatically measures several physical parameters of constituent particles in aggregates and agglomerates (or present as single particles) from EM images.

In this validation study, the measurement of four physical parameters was evaluated: the Feret-min as a measure of the minimal external dimension, the ECD as a size parameter that allows comparing measurement results with other techniques, the aspect ratio as a shape parameter, and the solidity as a surface topology parameter. All parameters are defined in S3.1.1. Use of the selected parameters is justified by the correlation matrix shown in Table S5. The validation study evaluated the measurement of the median value of the distributions of the four selected parameters.

The image analysis on the NM-300K particles was performed in "Default" mode, as the material consisted mainly of non-aggregated, near-spherical particles similar to those found in stable aqueous colloids such as ERM-FD100.

Table S5. Correlation matrix for measured physical parameters of NM-300K.

	Feret Min	ECD	Feret Max	Aspect Ratio	Elongation	Convexity	Solidity
Feret Min	1.00						
ECD	0.99	1.00					
Feret Max	0.96	0.99	1.00				
Aspect Ratio	0.17	0.05	0.06	1.00			
Elongation	0.15	0.03	0.10	0.83	1.00		
Convexity	0.19	0.21	0.25	0.10	0.11	1.00	
Solidity	0.17	0.14	0.06	0.34	0.32	0.53	1.00

S3.3. Materials

The validation study was performed on NM-300K material, obtained from the nanomaterial repository of the European Commission's Joint Research Centre, Institute for Health and Consumer Protection (JRC, Ispra, Italy). The material was received as a dispersion of silver nanoparticles in a glass vial, sealed under argon atmosphere with a nominal silver content of 10% *w/w*. Figure S5 shows a representative TEM image of the material.

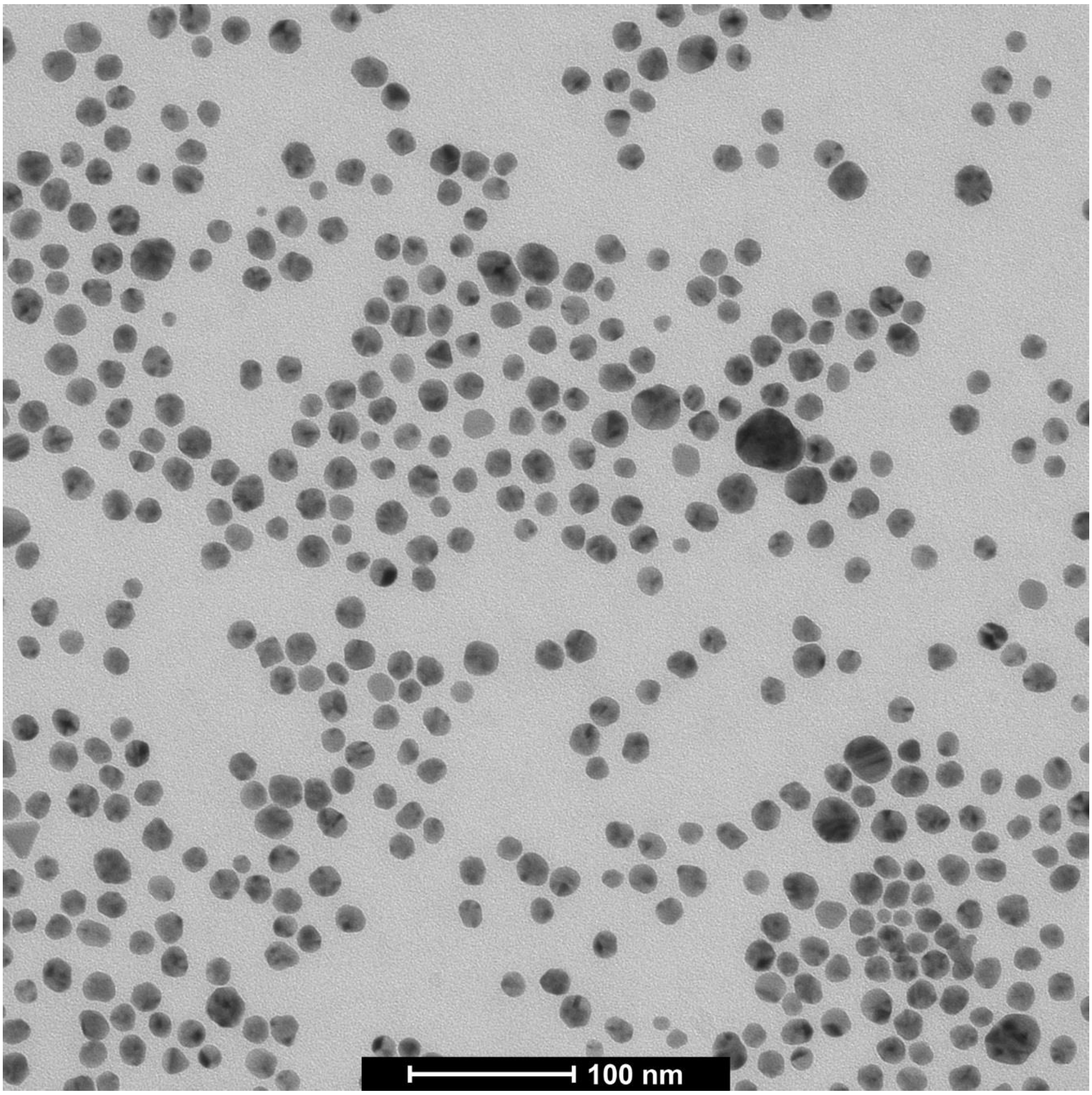

Figure S5. Representative micrograph of the NM-300K material. Bar: 100 nm.

S3.4. Experimental Design

The experimental design described by Verleysen et al. [11] was followed. The measurement uncertainties associated with the quantitative TEM measurement of the median Feret-min, median ECD, median aspect ratio, and median solidity were estimated using a top-down approach. A set of 150 images was generated by performing measurements on five days within one week. On each day, three TEM specimens were prepared from one vial and imaged by TEM. From each specimen, 10 images were recorded systematically and randomly over the grid surface.

S3.5. SOPs

The methodology to characterize the selected materials consisted of a combination of three SOPs:

- Sample preparation: SOP/NANoREG/D2.10/TEMSpePrep: "Preparation of EM-grids containing a representative sample of a dispersed NM"[12];
- Imaging: SOP/NANoREG/D2.10/TEMIma: "Transmission electron microscopic imaging of nanomaterials" [13];

- Image analysis: SOP "Measurement of the minimal external dimension of the primary particles of particulate materials from TEM images by the NanoDefine ParticleSizer software".

S3.6. Validation Parameters

S3.6.1. Working Range

The working range was determined as described by Verleysen et al. [11]. The selected magnification of 68,000× resulted in a limit of detection of 0.16 nm, a lower limit of quantification of 1.6 nm, and an upper limit of quantification of 66 nm. The useful working range of the applied TEM and CCD camera configuration was defined by the lower and upper size quantification limits, and resulted in a factor of about 40.

S3.6.2. Selectivity

To avoid subjectivity in the selection of particles by the microscopist, the SOP for TEM imaging specifies that the micrographs are taken randomly and systematically, at 10 positions pre-defined by the microscope stage and evenly distributed over the entire grid area. When the field of view was not suitable (e.g., because it was obscured by a grid bar or contained an artifact), the stage was moved sideways to the nearest suitable field of view. Micrographs of 10 regions on the grid were recorded with a 4 × 4 k Eagle CCD camera (FEI, Eindhoven, the Netherlands) using the TEM imaging & analysis (TIA) software (FEI, Eindhoven, The Netherlands). For the given microscope and camera configuration, a magnification of 68,000 times resulted in micrographs with a field of view of 660 nm by 660 nm.

Before particles are detected, the software creates a noise-reduced and background-subtracted image to avoid detecting contamination in the background. Afterwards, most particles of the selected material were detected by the software.

S3.6.3. Ruggedness and Robustness

Ruggedness against the number of measured particles—The ruggedness of the method and the validation study were evaluated against variation in the number of measured particles by determining the uncertainty associated with within-lab intermediate precision (u_{IP}) (see Section S3.6.4) of the quantitative TEM analysis from sub-datasets of measurements as a function of the number of analyzed particles.

For NM-300K, the relation between the number of measured particles and the measurement uncertainty of the Feret-min measurement was reported in the CEN/TS 17273 Guidance on detection and identification of nano-objects in complex matrices [14]. The measurement uncertainty reduces only marginally when the number of particles is higher than 100, as observed earlier for TEM analysis of near-monomodal, near-spherical silica particles [11,14].

Robustness against small variations in the image analysis settings—The effect of introducing small variations in the image analysis settings was generally small. However, the detection of some particles showing a high degree of poly-crystallinity and a low object-to-background intensity was influenced by these small variations. Mostly, artefacts were introduced when the Min. OTB intensity difference and smoothing factor were altered. Increasing the Min. OTB intensity difference value too much led to fewer detected low-contrast particles, and a chosen smoothing factor that was too high generally introduced less correctly detected particles. Optimizing the local threshold by the circular window radius option (at 80 px) allowed the correction of some wrongly segmented particles in default mode (Figure S6).

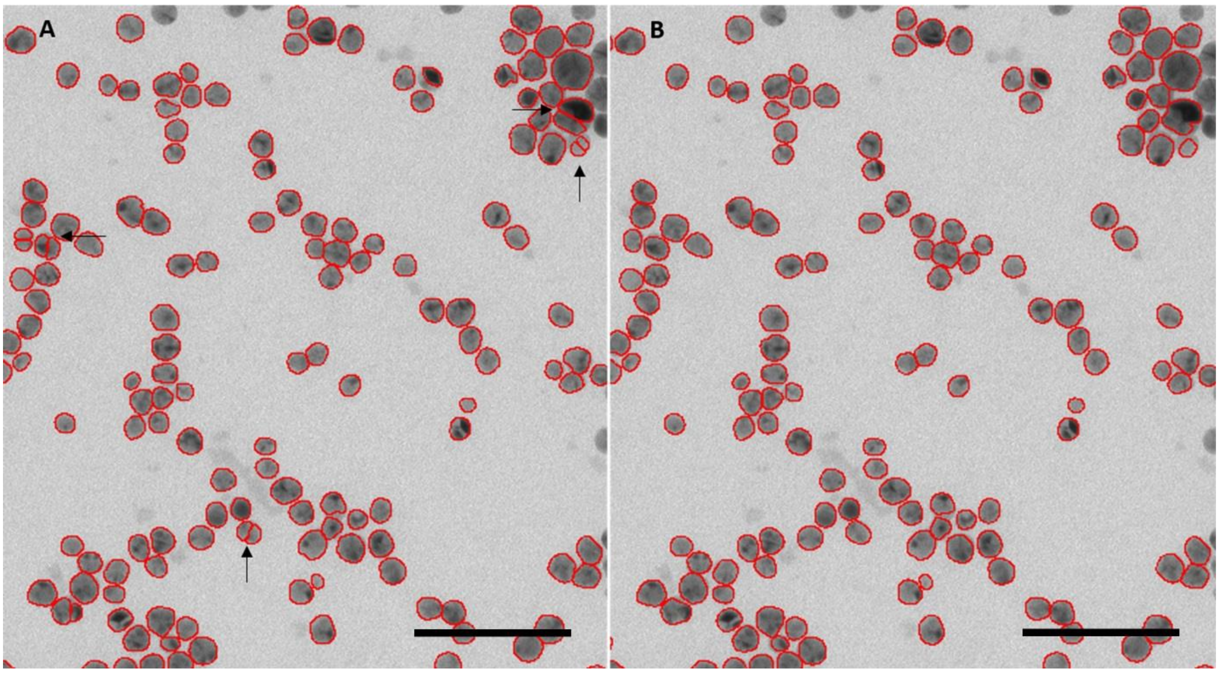

Figure S6. TEM image analyzed by the ParticleSizer software showing some wrongly detected particles indicated by black arrows (**A**) and the same image analyzed under similar conditions including a circular window radius at 80 px (**B**). Bar: 100 nm.

S3.6.4. Precision

The measurement uncertainties associated with the quantitative TEM measurement of the median Feret-min, ECD, aspect ratio, and solidity were estimated using a top-down approach as described by Verleysen et al. [11]. The image analysis settings were optimized on a few images. The selected image analysis settings were then applied to the 150 images, in sets of 10 images. Finally, 15 median Feret-min, ECD, aspect ratio, and solidity values were obtained and used for the ANOVA. Data processing was performed using MS Excel.

Table S6 summarizes the results obtained for the uncertainties associated with repeatability, u_r, within-day variation, u_{day}, and intermediate precision, u_{IP}. These uncertainties were determined as described by Verleysen et al. [11]. The determined u_{IP} values were smaller than 5% for both the Feret-min and the ECD. Similar values for the u_{day} and u_r were obtained. The determined u_{IP} values were smaller than 1% for both the aspect ratio and the solidity. The values of the u_{day} and u_r contributed equally to the u_{IP} for measurement of the median aspect ratio. However, the u_{day} was the largest contribution to the u_{IP} for measurement of the median solidity.

S3.6.5. Trueness and Uncertainty Associated with Trueness

For the median Feret-min, median ECD, median aspect ratio, and median solidity of NM-300K, no certified reference values are currently available. In these cases, the uncertainty associated with trueness was estimated based on other existing certified reference values. For ERM-FD100, a certified reference value, C_{CRM}, and its uncertainty, $u_{t,CRM}$, are available for the modal ECD value obtained by electron microscopy. It was assumed that the $u_{t,CRM}$, of modal ECD measurements originating from the CRM was a good estimate for the uncertainty associated with the trueness of the median Feret-min and median ECD of NM-300K. Since no reference values were available for the median aspect ratio and median solidity measurements of NM-300K, the uncertainty budget associated with trueness for these shape and surface topology parameters was calculated in the same way as for the size parameters. This is not ideal, since the correlation matrix shown in Table S5 showed only a minor correlation between these parameters. Table S6 summarizes the results obtained for the uncertainties associated to trueness, u_t. These uncertainties were determined as described by Verleysen et al. [11].

The trueness (bias) was not assessed, because no certified reference values were available for NM-300K.

S3.6.6. Combined and Expanded Measurement Uncertainty

The uncertainty contributions explained above were combined in the method's full uncertainty budget as described by Verleysen et al. [11]. Comparable U_{cx} values were obtained for the median Feret-min and median ECD. In absolute numbers, the obtained U_cx values of 11.8% for the median Feret-min and 11.3% for the median ECD give absolute uncertainties of 1.8 nm and 1.9 nm, respectively. The obtained U_cx values of 7.0% and 6.7% for the median aspect ratio and the median solidity correspond to absolute values of 0.08 and 0.07, respectively. It is expected that the obtained uncertainty budgets are a realistic representation of the expected measurement uncertainties. The trueness uncertainty was in all cases the largest contribution to the uncertainty budget.

Table S6. Summary of the different uncertainty contributions to the combined and expanded measurement uncertainties, for the median Feret-min, ECD, aspect ratio, and solidity obtained by the Default mode.

	**Feret-min**	**ECD**	**Aspect Ratio**	**Solidity**
Mean: Xm	15.4 nm	16.4 nm	1.08	0.99
Standard deviation: sd	0.5 nm	0.5 nm	0.01	0.0005
Uncertainty associated with repeatability: u_r	2.4%	2.2%	0.5%	0.05%
Uncertainty due to day-to-day variation: u_{day}	2.5%	2.3%	0.6%	0.0%
Uncertainty associated with intermediate precision: u_{IP}	3.4%	3.2%	0.8%	0.05%
Uncertainty associated with calibration: u_{cal}	0.2%	0.2%	0.2%	0.2%
Uncertainty associated with trueness: u_{tr}	4.8%	4.6%	3.4%	3.4%
Combined uncertainty: $u_c(x)$	5.9%	5.7%	3.5%	3.4%
Measurement uncertainty (k = 2): U_{cx}	11.8%	11.3%	7.0%	6.7%

S3.7. Conclusion

The validation study showed that the ParticleSizer software succeeded in obtaining precise characterization results. Expanded measurement uncertainties (U_cx) of 11.8%, 11.3%, 7.0%, and 6.7% were obtained for measurement of the mean median Feret-min, mean median ECD, the mean median aspect ratio, and mean median solidity by quantitative TEM, respectively.

In general, u_r did not significantly differ from u_{day}. u_t was in all cases the largest contribution to the uncertainty budget. More than 100 particles were measured in each sample to ensure optimal determination of u_{IP}. An effect of small variations in the ParticleSizer settings was only found in particles showing a high degree of poly-crystallinity and a low object-to-background intensity. Detection of artefacts was most pronounced when the Min. OTB intensity difference and smoothing factor were altered. However, the effect on the median Feret-min value was relatively small, and was generally not larger than a few nanometers. Besides being applicable to NM-300K, it is assumed that the measurement uncertainties of this validation study can be used to approximate the measurement uncertainties of other materials with similar properties analyzed by quantitative TEM.

S4. Variation Sources Among Replicate Analyses of NF@-Ag-005

During validation of Ag-005 (E174, 2 mm flakes) additional analyses were performed to verify the sources of repeatability variation. Each of the three replicates was in turn diluted in triplicate, and each dilution was measured twice by spICP-MS to differentiate the variation introduced by the measurement, the dilution before analysis, and the sample itself. Analysis results are given in Table S7. The repeated analyses of the same dilution and the repeated dilutions of the same replicate all

gave similar results. Only between the three independently prepared replicates (prepared and analyzed under repeatability conditions) did the results differ.

Table S7. Median equivalent spherical diameter (ESD) and the particle number (C_p) and mass (C_m) concentration in each of two measurements of triplicate dilutions of three independent replicates of E174 flakes (Ag-005).

		**ESD (nm)**		**C_p (× 10^{15} kg^{-1})**		**C_m (g/kg)**	
	Measurement Dilution	1	2	1	2	1	2
REP 1	1	27	27	9.1	9.0	3.2	2.6
	2	28	27	8.5	8.4	2.9	2.6
	3	30	30	7.8	8.3	3.1	3.0
REP2	1	22	23	5.5	5.8	1.4	1.5
	2	21	23	6.7	6.1	1.4	1.4
	3	22	22	7.0	6.2	1.7	1.2
REP 3	1	27	28	8.2	7.1	2.6	2.5
	2	29	30	7.3	7.2	2.5	2.6
	3	29	30	6.8	7.3	2.3	3.0

S5. Estimation of Uncertainty Related to Sample Preparation, Analysis, and Data Interpretation

To estimate the uncertainty related to sample preparation, the methodological procedure was segmented according to different stages, each having their own errors:

sampling → dispersion preparation → dilution → measurement → data interpretation.

The measurement uncertainty related to the spICP-MS analysis of NM-300K covers dilution, measurement, and data interpretation (= $u_{analysis}$). The uncertainty related to the analysis of E-174 containing products covers the same stages ($u_{analysis}$) plus sampling and dispersion preparation ($u_{sample\ preparation}$). Hence, the measurement uncertainty can be segmented in

$$u_c = \sqrt{u_{sample\ preparation}^2 + u_{analysis}^2} \qquad k = 1. \tag{8}$$

The uncertainty related to sample preparation can then be calculated as:

$$u_{sample\ preparation} = \sqrt{u_c^2 - u_{analysis}^2} \qquad k = 1, \tag{9}$$

or

$$u_{sample\ preparation} = \sqrt{u_{products}^2 - u_{NM-300K}^2} \quad k = 1. \tag{10}$$

The uncertainty related to the analysis of E-174 covers data interpretation at its limits on top of sample preparation and analysis. By adding a term representing data interpretation uncertainty to Equation (8) ($u_{data\ interpretation}$), the uncertainty added by a difficult data interpretation can be estimated as well:

$$u_c = \sqrt{u_{sample\ preparation}^2 + u_{analysis}^2 + u_{data\ interpretation}^2} \quad k = 1 \tag{11}$$

or

$$u_{data\ interpretation} = \sqrt{u_c^2 - u_{sample\ preparation}^2 - u_{analysis}^2} \quad k = 1, \tag{12}$$

$$u_{data\ interpretation} = \sqrt{u_{E174}^2 - u_{products}^2} \qquad k = 1. \tag{13}$$

Table S8 summarizes the estimated uncertainties related to sample preparation, analysis, and data interpretation at its limits. Since the uncertainties depend on the number of replicates that are

analyzed, the uncertainties were calculated for a single replicate analysis and a triplicate analysis, performed under repeatability conditions.

Table S8. Uncertainties associated with sample preparation, spICP-MS analysis, and data interpretation for sizing AgNP by means of a single replicate analysis or a triplicate analysis under repeatability conditions.

	U (%; k = 2)	u (%; k = 1)	$u_{sample\ preparation}$ (%; k = 1)	$u_{analysis}$ (%; k = 1)	$u_{data\ interpretation}$ (%; k = 1)
		Single Replicate Analysis			
NM-300K	13.3	6.7	-	6.7	-
Products containing E174	19	9.5	6.8	6.7	-
E174	21.5	10.8	6.8	6.7	5.0
		Triplicate Analysis Under Repeatability Conditions			
NM-300K	13	6.5	-	6.5	-
Products Containing E174	16	8.0	4.7	6.5	-
E174	20	10	4.7	6.5	6.0

Reference

1 Pace, H.E.; Rogers, N.J.; Jarolimek, C.; Coleman, V.A.; Higgins, C.P.; Ranville, J.F. Determining transport efficiency for the purpose of counting and sizing nanoparticles via single particle inductively coupled plasma mass spectrometry. *Anal Chem*, **2011**, *83*, 9361–9369.

2 *Compendium of Chemical Terminology (the "Gold Book")*, 2nd ed; McNaught, A.D., Blackwell, W.A. eds.; Scientific Publications: Oxford, UK, 1997.

3 *Eurachem Guide: The Fitness for Purpose of Analytical Methods – A Laboratory Guide to Method Validation and Related Topics*, 2nd ed.; Magnusson, B., Örnemark, U. eds.; *ISBN 978-91-87461-59-0.* Available from https://www.eurachem.org (accessed on 20 August 2019).

4 Cornelis, G.; Hassellöv, M. A signal deconvolution method to discriminate smaller nanoparticles in single particle ICP-MS. *J. Anal. Spectrom*. **2014**, *29*, 134–144.

5 *ISO. ISO 14644-6: Cleanrooms and Associated Controlled Environments-Part 6: Vocabulary, 2007*; International Organization for Standardization: Geneva, Switzerland, 2007.

6 *ISO. Technical Specification ISO/TS 27687: Terminology and Definitions for Nano-Objects-Nanoparticle, nanofibre and nanoplate in Nanotechnologies*; International Organization for Standardization: Geneva, Switzerland, 2008.

7 *ISO. Technical Specification ISO/TS 16195: Nanotechnologies—Guidance for Developing Representative Test Materials Consisting of Nano-Objects in Dry Powder Form*; International Organization for Standardization: Geneva, Switzerland, 2013.

8 Wagner, T.; Lipinski, H.G. IJBlob: An imagej library for connected component analysis and shape analysis. *J. Open Res. Softw.* **2013**, *1*, e6. doi:10.5334/jors.ae

9 Wagner, T. ParticleSizer thorstenwagner/ij-particlesizer: v1.0.9. *https://zenodo.org/record/820296#.XAZ9XtKi00* (accessed on 20 August 2019)

10 Rausher, H.; Roebben, G.; Mech, A.; Gibson, N.; Kestens, V.; Linsinger, T.P.J.; Sintes, J.R. *An Overview of Concepts and Terms used in the European Commission's Definition of Nanomaterial*; EUR 29647 EN, European Commission, JRC, Ispra, Italy, 2019. ISBN 978-92-79-99660-3.

11 Verleysen, E; Wagner, T.; Lipinski, H.G.; Kägi, R.; Koeber, R.; Boix-Sanfeliu, A.; De Temmerman, P.J.; Mast, J. Evaluation of a TEM based approach for size measurement of particulate materials. *Materials* **2019**, *12*, 2274.

12 NANoREG D2.10 SOP 01 Preparation of EM-grids containing a representative sample of a dispersed NM. https://www.rivm.nl/documenten/nanoreg-d210-sop-01-preparation-of-em-grids-containing-representative-sample-of (accessed on 20 August 2019)

13 Standard Operating Procedure. https://www.rivm.nl/sites/default/files/2018-11/NANoREG%20D2.10%20SOP%2002%20Transmission%20electron%20microscopic%20imaging%20of%20nanomaterials.pdf (accessed 20 August 2019)
14 CEN. CEN/TS 17273, Nanotechnologies-Guidance on Detection and Identification of Nano-Objects in Complex Matrices; European Committee for Standardization: Brussels, Belgium, 2018. ISBN 978-0-539-00281-2.

© 2019 by the authors. Submitted for possible open access publication under the terms and conditions of the Creative Commons Attribution (CC BY) license (http://creativecommons.org/licenses/by/4.0/).
